# Missense variants in *MC4R* gene are associated with obesity in cats

**DOI:** 10.1007/s11259-025-10700-4

**Published:** 2025-03-04

**Authors:** Salah Aldin Mousa Basha, Iraz Akis

**Affiliations:** Department of Veterinary Biochemistry, Faculty of Veterinary Medicine, Istanbul University-Cerrahpasa, Buyukcekmece Campus, Istanbul, 34500 Türkiye

**Keywords:** Body condition score, Body mass index, Cat, *MC4R* gene, Obesity, Polymorphism

## Abstract

Obesity stands out as the most common multifactorial nutritional problem affecting domestic cats. According to studies, the prevalence of overweight or obese cats varies between 11.5% and 63%. Various factors such as breed, age, gender, reproductive status, owner-pet relationship, diet type, and environmental factors have been identified as potential risk factors for the development of obesity in cats. Among the genes involved in regulating energy balance, one of the prominent genes is melanocortin-4 receptor gene (*MC4R*). A specific missense variant in the feline *MC4R* gene (c.92 C > T) has been associated with overweight in diabetic domestic shorthaired cats. In this study, it was aimed to determine the polymorphisms in *MC4R* gene in random bred cats and cats belonging to a registered breed in Turkey and to investigate their relationship with obesity. Blood samples from 30 obese and 20 non-obese cats were collected into sterile vacuum EDTA tubes. Exon 1 of the *MC4R* was amplified and sequenced. As a result of DNA sequence analysis, we identified a total of six SNPs in the feline *MC4R* gene, four of which were found for the first time in this study. As a result of comparing allele frequencies in obese and non-obese cats, a significant relationship was found between SNP rs783632116 and obesity. The results of regression analyses evaluating the effects of SNP genotypes, sex and infertility status on feline Body Mass Index (fBMI) indicated that non-synonymous SNPs rs783632116, ss11356259660 and ss11356259661 were significantly associated with fBMI.

## Introduction

Obesity is a result of overall excess of energy intake, which is especially common in sterilized and middle-aged cats. Obesity predisposes cats to a variety of metabolic and clinical disorders, including insulin resistance, Diabetes mellitus, lameness and skin disease (Hoelmkjaer and Bjornvad 2014). Pets are defined as obese when their body weight exceeds 30% of optimal (German 2006). According to a study conducted in the USA, 1/3 of domestic cats and dogs fall into the overweight category. The primary reason for this situation is that the animals have been removed from their natural habitats (Chandler et al. 2017).

There are many studies examining the factors that cause obesity, such as genetics, diet, sterilization and physical activity. The higher risk of obesity in certain cat breeds indicates that the disease is also related to genetic factors (Chandler et al. 2017). Evidence from recent studies suggest that obesity is caused by the interaction of environmental and genetic factors (Jerjen et al. 2023).

According to the results of the genome-wide association studies in humans, the most remarkable genes, associated with an increased risk of obesity, are MC4R, BDNF, LEP, LEPR and FTO (Wardle et al. 2008; Dall’aglio et al. 2012; Farooq et al. 2021). Melanocortin-4 receptor (MC4R) and brain-derived neurotrophic factor (BDNF) have major roles in the leptin-proopiomelanocortin pathway. This pathway is involved in the regulation of energy homeostasis, and therefore regulates body weight and overall metabolism (Farooq et al. 2021).

In previous studies, one missense (c.92 C > T, Proline31Leusine) variation was identified in the coding region of the feline *MC4R* gene. In a study conducted on overweight and underweight diabetic cats, the frequency of CC homozygous (c.92 C > T) individuals was determined to be 55% in overweight cats. The C allele has been suggested to be a risk factor for obesity in diabetic cats (Forcada et al. 2014).

In this study, we determined novel variants in the feline *MC4R* gene in random bred cats and pedigreed cats in Turkey, in addition to c.92 C > T polymorphism. Furthermore, we analysed the association between these variants and body condition score and body mass index.

## Materials and methods

### Samples

The study was conducted at the Internal Medicine Clinic of Istanbul University-Cerrahpasa, Research and Practice Animal Hospital. Blood samples from 30 obese and 20 non-obese cats, all non-diabetic, were collected. Body condition score (BCS) was calculated according to the routinely used 9-point BCS system (Laflamme 1997). Feline body mass index (fBMI) was calculated by dividing body weight by the length from the top of the patella to the end of the calcaneus (BW/PCL) (Kawasumi et al. 2016). The study was approved by the “Istanbul University-Cerrahpasa, Ethics Board of Faculty of Veterinary Medicine” with the verdict number 2022/44.

### DNA extraction, partial *MC4R* amplification and sequencing

Genomic DNA was isolated from whole blood samples using commercial kits (DNA Isolation Kit for Mammalian Blood, Roche Applied Science). A 609 bp part of the exon 1 of the feline *MC4R* gene was amplified using F: 5’- ACCTGACCCGAGAGATCGAA − 3’ and R: 5’- TGAACAAAACGCCCGAAACC − 3’ primer pair. PCR amplifications were performed in a reaction volume of 25 µl using 2 µl 10XPCR buffer (100 mM KCl, 20 mM Tris HCl (pH 8.0), 0.1 mM EDTA, 0.5 mM PMSF, 1 mM DTT, 50% glycerol), 2.5 mM MgCl_2_, 50–100 ng genomic DNA, 100µM dNTP, 10 pmol of each primer and 1 U Taq polymerase. PCR was carried out as follows: Initial denaturation at 94 °C for 5 min; 30 cycles of denaturation at 94 °C for 60 s, annealing at 60 °C for 60 s, elongation at 72 °C for 60 s; and a final extension at 72 °C for 10 min. PCR products were checked by running 2% agarose gel electrophoresis. Amplicons were sequenced by using an ABI-3100 sequencer (PE Biosystems, Germany) and the BigDyeTM terminator cycle sequencing kit (ThermoFisher Scientific, USA), after purification with ExoSAP-ITTM PCR Product Cleanup kit (ThermoScientific, USA).

### Determination of sequence variations and in Silico analysis

The sequences of the feline *MC4R* gene from all the samples were aligned by using ClustalW program in the MEGA 11 software (Tamura et al. 2021). Sequences were compared to each other and the cat *MC4R* gene (GenBank ID: NC_058379.1) reference sequence at the National Center for Biotechnology Information. The open reading frame region was determined on the protein sequence (ID: M3W634) at UniProt (The UniProt Consortium 2023). The influence of non-synonymous SNPs on feline MC4R protein were evaluated by using PANTHER and PolyPhen-2 programmes.

### Statistical analysis

The sample size was determined using G*Power software (version 3.1.9.4) (Faul et al. 2007). A power analysis was performed with a 0.05 significance level and 0.8 power. Allele and genotype frequencies of the polymorphisms were calculated using IBM SPSS 25.0 program. Risk alleles for obesity were determined by cross-tabulation analysis in SPSS 25.0. Linear regression analysis was conducted to examine the effects of SNPs, gender and sterilization status on fBMI. Fisher’s exact test was performed to calculate the significance of the association.

## Results

We identified a total of six SNPs in the cat MC4R gene, four of which were identified for the first time in this study. The genetic polymorphisms determined in this study are available in the European Variation Archive under project accession ID PRJEB72817. Allele and genotype frequencies were presented in Table 1.

**Table 1:** Allele and genotype frequencies of the SNPs in feline *MC4R* gene

SNP	Groups^a^	Allele Frequency (%)		Genotype Frequency (%)			χ2^b^
rs783632116		T	C	TT	TC	CC	
	Underweight	72.22	27.78	55.5	33.33	11.11	
	Normal	86.36	13.64	72.72	27.27	0	
	Obese	48.33	51.67	30.00	36.66	33.33	
	Total	61	39	44.00	34.00	22.00	8.540
ss11356259660		G	A	GG	GA		
	Underweight	100	0	100	0		
	Normal	100	0	100	0		
	Obese	98.33	1.67	96.66	3.34		
	Total	98.00	2.00	98	2		0.680
ss11356259659		C	T	CC	TC	TT	
	Underweight	100	0	100	0	0	
	Normal	90.91	9.09	90.91	0	9.09	
	Obese	98.33	1.67	96.66	3.34	0	
	Total	97	3	96.00	2.00	2.00	4.258
ss11356259658		C	T	CC	TC	TT	
	Underweight	100	0	100	0	0	
	Normal	90.91	9.09	90.90	0	9.09	
	Obese	98.33	1.67	96.66	3.34	0	
	Total	97	3	96.00	2.00	2.00	4.258
ss11356259661		G	A	GG	GA	AA	
	Underweight	100	0	100	0		
	Normal	100	0	100	0		
	Obese	93.33	6.67	86.66	13.33		
	Total	96	4	92.00	8.00		2.899
rs785927510		C	T	CC	TC	TT	
	Underweight	88.89	11.11	88.88	0	11.11	
	Normal	95.45	4.55	90.90	9.09	0	
	Obese	100	0	100	0	0	
	Total	97	3	96.00	2.00	2.00	8.228

^a^Underweight: BCS 1–3, Normal: BCS 5, Obese: BCS 7–9

^b^Pearson’s chi-square test for assessing Hardy-Weinberg equilibrium

Three SNPs were non-synonymous. The first one was a T/C substitution (rs783632116) identified by Forcada et al. (2014) and leads to an Leucine31Proline (L31P) substitution. The other two SNPs were SNPs ss11356259660 and ss11356259661 and result in Alanine68Threonine (A68T) and Glutamic acid100Lysine (E100K) substitution, respectively.

The effects of amino acid changes in MC4R protein were evaluated by PolyPhen-2 and PANTHER programmes. L31P was predicted as “benign” (PolyPhen-2 score:0.000) and “probably benign” (Pdel score:0.13), A68T was predicted as “probably damaging” by both tools (PolyPhen-2 score:0.998, Pdel score:0.74). For the E100K replacement, there are incompatible results between the two bioinformatics tools. According to PolyPhen-2, this change was predicted “possibly damaging” (PolyPhen-2 score:0.803), while PANTHER predicted it as “probably benign“(Pdel score:0.13).

The average body condition score of the obese cat group was calculated as 8.27. The average body condition score of the non-obese cats was 4.2. The relationship between SNPs and obesity is given in Table 2. As a result of the comparison of allele frequencies in obese and non-obese cats, a significant association was found between SNP rs783632116 and obesity. The results of regression analyses evaluating the effects of SNP genotypes, sex and infertility status on fBMI indicated that SNPs rs783632116, ss11356259660 and ss11356259661 were significantly associated with fBMI (Table 3). SNP rs783632116 CC genotype was associated with higher fBMI than TT genotype, while SNP ss11356259660 and SNP ss11356259661 GA heterozygous genotypes were associated with higher fBMI than GG homozygous genotypes. For the first one, it can be suggested that the C allele is the risk allele, while for the latter two, the wild type homozygous genotypes is the protective genotype.

**Table 2:** Association between the SNPs in feline *MC4R* gene and obesity

SNP	Position^a^	Allele^b^	MAF^c^	OR^d^ (95% CI)	*P* ^e^
rs783632116	D3:78.474.087 (D3:80794589 in previous assembly)	T/C	0.39	4.276 (1.695–10.789)	0.002*
ss11356259660	D3:78.473.977	G/A	0.02	0.976 (0.930–1.024)	0.410
ss11356259659	D3:78.473.888	C/T	0.03	0.322 (0.028–3.676)	0.562
ss11356259658	D3:78.473.882	C/T	0.03	0.322 (0.028–3.676)	0.562
ss11356259661	D3:78.473.881	G/A	0.04	1.071 (1.001–1.146)	0.148
rs785927510	D3:78.473.876 (D3:80794378 in previous assembly)	C/T	0.03	0.925 (0.847–1.010)	0.061

*p < 0,01

^a^Position at the NCBI RefSeq assembly GCF_018350175.1

^b^Major/minor allele on minus strand

^c^Minor allele frequency

^d^Odds ratios

^e^Test for allele frequency differences between non-obese and obese cats using Fisher’s Exact Test

**Table 3:** Effect of sex, sterilization status and *MC4R* SNPs on feline body mass index

Factors	Groups	B	SE	Stand. B	95% CI for B	*P*-value^a^
Sex	Female-Male	− 4.65	3.34	− 0.371	− 0.9115 – − 0.1688	0.172
Sterilization	No-Yes	− 7.28	3.45	− 0.582	− 1.1398 – −0.0239	0.041*
rs783632116	TC – TT	2.23	3.96	0.178	0.4628–0.8198	0.576
	CC – TT	9.84	4.34	0.878	0.0836–1.4894	0.029*
ss11356259660	GA – GG	24.35	10.37	1.947	0.2673–3.6258	0.024*
ss11356259659	TC – TT	3.21	14.86	0.256	2.1505–2.6634	0.830
	CC – TT	3.23	10.73	0.258	1.4787–1.9953	0.765
ss11356259658	TC – TT	2.67	11.85	0.213	1.7054–2.1320	0.823
	CC – TT	3.19	10.73	0.255	1.4821–1.9919	0.768
ss11356259661	GA – GG	19.45	5.92	1.555	0.5962–2.5129	0.002**
rs785927510	TC – TT	− 4.39	14.51	− 0.351	− 2.7018–1.992	0.764
	CC – TT	2.50	10.63	0.200	1.5210–1.9206	0.815

*p < 0.05

**p < 0.01

^a^Fisher’s exact test to assess the effects of sex, sterilization status and *MC4R* SNPs on feline Body Mass Index

## Discussion

The studies on *MC4R* gene in cats are limited. In a study conducted by Forcada et al. (2014) on domestic shorthaired cats and Burmese cats in UK, a missense variant c.92 C > T was determined. The results showed that the C allele was associated with DM only in overweight cats. In another study on domestic shorthaired cats in Switzerland, no statistically significant relationship was found between this SNP and BCS and body fat percentage (Jerjen et al. 2023).

Contrary to the previous studies, in the current study MC4R: c.92 C > T (rs783632116) polymorphism was found to be significantly associated with BCS and fBMI in non-diabetic cats. Additionally, two novel SNPs, c.202G > A (ss11356259660) and c.298G > A (ss11356259661), were found to be associated with fBMI.

MC4R is mainly expressed in the hypothalamus and is part of the pathway regulating appetite and energy balance. In a state of positive energy balance, MC4R is stimulated by alpha-melanocyte stimulating hormone, leading to appetite suppression (Wallis and Raffan 2020). Variants in the *MC4R* gene may have an effect on the function of the protein and might cause an increase in body weight.

The missense MC4R: c.92 C > T polymorphism results in L31P change. The effect of this MC4R protein change was predicted as bening and probably bening by bioinformatics tools. Considering the findings that the C allele was identified as a risk allele for obesity and that the CC genotype was associated with higher fBMI, bioinformatic predictions indicate that these effects are not due to a structural change in the MC4R protein. However, due to proline’s special side chain, leucine-proline change might affect protein-protein interactions in metabolic pathways involving MC4R. Studies in humans have revealed that mutations in genes encoding transmembrane proteins are generally associated with diseases. It is reported that the majority of disease-associated mutations result in a leucine-proline change (Molnar et al. 2016).

The novel SNP c.202G > A (ss11356259660) identified in the current study causes A68T substitution, which was predicted as probably damaging by bioinformatic tools. The statistically significant association between GA genotype and increased fBMI might be explained by this damaging effect on MC4R protein. Other novel SNP associated with fBMI is a non-synonymous polymorphism c.298G > A (ss11356259661) resulting in E100K change. In this study, GA genotype was found to be significantly associated with higher fBMI than GG. Two bioinformatic tools gave distinct results for the effect of glutamic acid-lysine substitution. While PolyPhen-2 predicted its effect as possibly damaging, PANTHER predicted it as probably bening. This difference between the tools may be due to the algorithm approach they use (Güvendi et al. 2024). Glutamic acid and lysine have different chemical properties, the former is an acidic amino acid and the latter is a basic amino acid. Substitution of these two chemically different amino acids might have effects both within the protein molecule and in the interaction between MC4R protein and other molecules.

In conclusion, results of this study suggest that the MC4R gene might be a candidate gene for obesity in cats. Three non-synonymous SNPs (rs78363632116, ss11356259660 and ss11356259661) in the MC4R gene were found to be statistically associated with BCS and/or fBMI in cats. Further studies on cat samples from different countries and breeds would shed light on the relationship between this gene and obesity and will be useful in revealing the molecular role of MC4R protein in energy metabolism in cats.

## Data Availability

The genetic polymorphisms determined in this study are available in the European Variation Archive under project accession ID PRJEB72817.
